# Unveiling the Mainland vs. Insular Variability of the *Eumerus barbarus* Species Group (Diptera: Syrphidae) in the Western Mediterranean Basin †

**DOI:** 10.3390/insects15040239

**Published:** 2024-03-29

**Authors:** Pablo Aguado-Aranda, Antonio Ricarte, Zorica Nedeljković, Martin Hauser, Scott Kelso, Lucía Sainz-Escudero, Jeffrey H. Skevington, María Ángeles Marcos-García

**Affiliations:** 1 Research Institute CIBIO (Centro Iberoamericano de la Biodiversidad), Science Park, University of Alicante, Ctra. San Vicente del Raspeig s/n, 03690 San Vicente del Raspeig, Alicante, Spain; ricarte24@gmail.com (A.R.); zoricaned14@gmail.com (Z.N.); marcos@ua.es (M.Á.M.-G.); 2 Plant Pest Diagnostics Centre California, Department of Food and Agriculture, 3294 Meadowview Road, Sacramento, CA 95832-1448, USA; phycus@gmail.com; 3 Canadian National Collection of Insects, Arachnids and Nematodes, Agriculture and Agri-Food Canada, K.W. Neatby Building, 960 Carling Avenue, Ottawa, ON K1A 0C6, Canada; scott.kelso@agr.gc.ca (S.K.); jhskevington@gmail.com (J.H.S.); 4 Museo Nacional de Ciencias Naturales (MNCN, CSIC), C/José Gutiérrez Abascal 2, 28006 Madrid, Spain; deliciasluciasainz@gmail.com

**Keywords:** hoverflies, Merodontini, barcode, genetic diversity, phenotypic variation, new species

## Abstract

**Simple Summary:**

Hoverflies, also known as flower flies, are a highly diverse group of two-winged flies with more than 6000 known species worldwide. This group of insects plays an important role in ecosystems, providing services such as plant pollination and pest control, among others. Regarding *Eumerus*, this hoverfly genus is one of the most speciose in the Palaearctic region, where new species are continually being discovered. Nevertheless, the great morphological variability of some species makes their identification and classification difficult. Therefore, the main aim of the present work was to assess the variability of the species of the *Eumerus barbarus* group in the western Mediterranean area under an integrative study (i.e., combining different techniques and data sources). We found high levels of morphological and genetic variability in two species of this group. Based on our findings, we described a new species from the island of Sardinia and provided the most comprehensive identification key for the males of this *Eumerus* species group from the western Mediterranean.

**Abstract:**

Comprising nearly 300 described species, *Eumerus* Meigen, 1822, is one of the most speciose syrphid genera worldwide, and its taxonomic diversity is remarkable in the Mediterranean basin. The *Eumerus barbarus* (Coquebert, 1804) group consists of four species in the western Mediterranean. Although the phenotypic variability of this species group has been commented on in previous studies, it has never been contrasted with molecular data. In the present work, the morphological variation found in 300+ specimens of this species group from the western Mediterranean is explored and tested against the COI mitochondrial DNA (mtDNA). The highest phenotypic disparity was found in *E. barbarus* and *Eumerus sulcitibius* Rondani 1868. The integrative approach has not revealed cryptic diversity within the species *E. barbarus* but in *E. sulcitibius*. As a result, a new species close to *E. sulcitibius* was discovered, *Eumerus sardus* Aguado-Aranda, Ricarte & Hauser sp. n., from Sardinia, Italy. The new insular species is here described, illustrated, and discussed. A total of twenty-three haplotypes of COI mtDNA were identified amongst the analyzed Mediterranean specimens of *E. barbarus*, whereas two and five haplotypes were distinguished in the Iberian specimens of *E. sulcitibius* and *Eumerus gibbosus* van Steenis, Hauser & van Zuijen, 2017, respectively. Moreover, the first known barcodes of *E. gibbosus* and *Eumerus schmideggeri* van Steenis, Hauser & van Zuijen, 2017 were obtained, and the distribution ranges of all species are mapped. An updated dichotomous key to the males of the *E. barbarus* group from the western Mediterranean is provided.

## 1. Introduction

The genus *Eumerus* Meigen, 1822 (Eristalinae: Merodontini), with 300+ described species and yet-undescribed species globally, is one of the largest syrphid genera worldwide [1]. This genus comprises species with a robust, black, or dark grey to brown body, sometimes with metallic reflections; a face without protuberances; an arista bare; an abdomen with three pairs of white, half-moon-shaped maculae on it (in most species); a wing vein M_1_ bent inwards; and a cell R_2+3_ open at wing margin, among other features [2,3]. It was originally distributed in the Old World [4,5], but some species have been introduced into the New World by human activity [6,7].

The first classification of *Eumerus* into species groups was conducted by Chroni et al. [8] based on COI mitochondrial DNA. Nowadays, eleven groups are distinguished within the genus [9], of which seven have also been defined morphologically (e.g., [10,11]). For instance, the *E. barbarus* group, which is the focus of the present study, was defined by van Steenis et al. [5] in order to accommodate species with a face densely white pollinose and covered with whitish yellow hairs; eyes with hairs; apex of the vertical triangle white pollinose; vertical triangle with two white pollinose maculae behind posterior ocelli; hind femur thickened, with long hairs ventrally; in males, eyes holoptic; hind trochanter with a triangular-like expansion; hind tibia with a ventral lamina posteriorly; and posterior surstylar lobe of genitalia elongated and hook-shaped apically. The latter authors also described two new species, of which one is an Iberian endemic, *Eumerus gibbosus* van Steenis, Hauser & van Zuijen, 2017, and other a North African endemic, *Eumerus schmideggeri* van Steenis, Hauser & van Zuijen, 2017. Therefore, four species are known to occur in the western Mediterranean: *E. barbarus*, *E. gibbosus*, *E. schmideggeri*, and *Eumerus sulcitibius* Rondani, 1868.

As for other Palaearctic syrphid genera with complex taxonomy such as *Chrysotoxum* Meigen, 1803, and *Merodon* Meigen, 1803 [12,13], the taxonomic diversity is far to be fully known for *Eumerus* [8]. In this regard, a pending issue is to learn how the observed phenotypic variation agrees with taxonomic diversity in *Eumerus*. In this type of analysis, an approach combining different sources of data (morphological, genomic, and morphometrics) provides evolutionary clarification (e.g., [14,15]). However, as for phenotypic features, molecular features can also be variable; for example, while low interspecific variation in COI was revealed for certain species of the *Eumerus tricolor* (Fabricius, 1798) group [16], high levels of intraspecific variability (same molecular marker) have been found in another six *Eumerus* species from the Mediterranean basin [17].

The Mediterranean basin is considered one of the most important biodiversity hotspots in the world and includes a vast mosaic of habitats with high species diversity [18]. Furthermore, geological and climatic histories facilitated species diversification, as a large percentage of biodiversity is endemic to the Mediterranean basin [19]. Concerning the Mediterranean islands, a high degree of endemicity is observed in these areas (e.g., [20]). At present, four species of *Eumerus* were only reported from islands in the Mediterranean area: *Eumerus minotaurus* Claußen & Lucas, 1988; *Eumerus olivaceus* Loew, 1848; *Eumerus sicilianus* van der Goot, 1964; and *Eumerus vandenberghei* Doczkal, 1996 [21]. A preliminary examination of material of the *E. barbarus* group from the islands and mainland from the western Mediterranean basin revealed a high morphological variability, mainly in *E. barbarus* and *E. sulcitibius*. Although previous studies have assessed variability using molecular markers for other Merodontini (e.g., [22]), few studies have assessed it in *Eumerus* (e.g., [16]). Therefore, the main aim of this work is to explore the phenotypic variability of the species of the *E. barbarus* group from the western Mediterranean basin in correlation with genetic data.

## 2. Materials and Methods

### 2.1. Study Area

We followed van Steenis et al. [5] in our concept of the “western Mediterranean basin”. Regarding the Ibero-Balearic area (mainland Portugal and Spain, plus Andorra, Gibraltar, and the Balearic Islands), two biogeographical regions are represented: Eurosiberian and the Mediterranean [23]. The first one is restricted to the northernmost areas, where mainly mountainous landscapes are found, while the Mediterranean includes a wide set of habitats [24].

We sampled all available material from the western Mediterranean region. Sampling campaigns took place in the Balearic Islands of Mallorca and Menorca and mainland Spain. Regarding the Eurosiberian region, the province of León (“Parque Nacional Picos de Europa”) was surveyed. Fieldwork in the Mediterranean region of the Iberian Peninsula was conducted in the provinces of Alicante (“Sierra de Aitana”), Almería (“Sierra de Gádor”), Cáceres, Madrid (“Parque Nacional Sierra de Guadarrama”), Salamanca, Valencia (“Sierra de Mariola”), and Zamora (see Supplementary Material S1).

### 2.2. Examined Material

As a result of the fieldwork, 253 new specimens of *Eumerus* were collected by hand net: 232 of *E. barbarus* (138 males and 94 females), 5 of *E. gibbosus* (2 males and 3 females), 1 male of *E. schmideggeri*, and 15 (14 males and 1 female) of *E. sulcitibius*. We also examined material deposited in the following collections: “Colección Entomológica de la Universidad de Alicante” (CEUA-CIBIO, Alicante, Spain), the Canadian National Collection of Insects, Arachnids and Nematodes (CNC, Ottawa, ON, Canada), California State Collection of Arthropods (CSCA, Sacramento, CA, USA), “Museo Nacional de Ciencias Naturales” (MNCN, Madrid, Spain), Zoological Research Museum Alexander Koenig (ZFMK, Bonn, Germany), and “Museum für Naturkunde der Humboldt Universität” (ZMHU, Berlin, Germany). In total, 336 adult specimens of five species of the *E. barbarus* group were studied in the present work: 303 specimens (170 males and 133 females) of *E. barbarus*; 5 specimens (2 males and 3 females) of *E. gibbosus*; 2 males of *E. schmideggeri*; 25 specimens (21 males and 4 females) of *E. sulcitibius*; and 2 males of *Eumerus* aff. *sulcitibius*.

All examined specimens were databased in an excel table. A unique collection bar-code label was assigned to the new material added to the CEUA-CIBIO collection. The format for the examined material lists followed Aguado-Aranda et al. [16], but different labels of the same specimen are separated by a double forward slash (//) and different sides of the same label are separated by a single forward slash (/). The information on the examined specimens is detailed in a Supplementary File (see Supplementary Material S1), but that of the new species is included in the main text document. Distribution maps of each species were produced with the software QGIS v3.22.16 [25]. For distribution maps of the most widespread species in the study area (i.e., *E. barbarus* and *E. sulcitibius*), only literature records from the Iberian Peninsula and the Balearic Islands [26,27,28,29,30,31,32,33,34] were considered in addition to the new ones.

### 2.3. Morphological Study

All material was identified with dichotomous keys [5,35]. Body length and the basoflagellomere ratio (length: width; see figure 4a of Aguado-Aranda et al. [36]) were measured with the software Leica Application Suite X (LAS X) ^®^ v3.0.4.16529. Male genitalia were dissected following Ricarte et al. [37] and then stored in glycerin in plastic microvials (pinned with the specimen). Photographs were taken with a Leica DFC 450 camera attached to a Leica M205 C binocular microscope. Male genitalia were hand drawn from printed photographs by the first author. Morphological terminology follows Thompson [38], except for the term “hair/s” (in replacement of “pilis/pile”), as well as the term “notopleural sulcus”, which follows Doczkal and Pape [39] for the new species description. Terminology for male genitalia follows Doczkal [40]. For those illustrations and tables cited from the literature “figure” and “table” (lower cases) are used, while “Figure” (upper cases) is used for those which are original to this work.

### 2.4. Molecular Study

DNA was extracted from one or two legs of 38 specimens (23 males and 15 females) of *E. barbarus*, 5 specimens (2 males and 3 females) of *E. gibbosus*, 1 specimen of *E. schmideggeri* (male), 4 specimens (3 males and one female) of *E. sulcitibius*, and 1 specimen of *Eumerus* aff. *sulcitibius* (male). DNA of three males of *E. barbarus* (collection codes CNC # DIPTERA 155809; 155826–27) was extracted from a wing. The NZY Tissue gDNA Isolation kit, following the manufacture’s protocol for animal tissues, was used for all specimens but three males of *E. barbarus* (codes previously indicated), one male of *Eumerus* aff. *sulcitibius*, and one male of *E. schmideggeri*. DNA of these specimens was extracted using the QIAGEN DNeasy Blood and Tissue kit, with some modifications to the manufacturer’s protocol, in the research laboratory of Jeffrey H. Skevington by the first author.

PCR amplification protocols of the 5′ (COI-5′) and 3′ (COI-3′) end regions of the Cytochrome c oxidase subunit I gene, and the COI-5′ fragments “A”, “B” and “C” for “older” specimens (collection date previous to the year 2000), followed Aguado-Aranda et al. [16]. Thermocycler conditions followed Vujić et al. [13] and Grković et al. [4] for COI-5′, and Chroni et al. [8], but with annealing at 48–49 °C, for COI-3′. The PCR profile for the COI-5′ fragments followed Aguado-Aranda et al. [16]. PCR products were visualized with an electrophoresis process in a 1–2% agarose gel. Purifications and sequencing reactions of COI-5′ fragments products followed Aguado-Aranda et al. [16]. The rest of the PCR products were purified and sequenced at Macrogen (Macrogen, Inc., Seoul, Republic of Korea).

The editing of the sequences was conducted with the program Sequencher v5.4.6 (Gene Codes Corporation 2017, Ann Arbor, MI). Then, COI-5′ and COI-3′ sequences of *Eumerus alpinus* Rondani, 1857, *E. minotaurus*, *Eumerus sogdianus* Stackelberg, 1952, and at least one specimen of each species of the *E. barbarus* group available at the public repository GenBank were downloaded (see Supplementary Material S2). This was performed to obtain a geographical and molecular representation of each species in the analyses. Alignments were performed manually and checked with the program AliView v1.25 [41]. The final COI-5′ and COI (COI-5′+COI-3′) matrices had lengths of 554 and 1134 bp, respectively.

Molecular analyses with 1000 replications were conducted using the Maximum Likelihood Composite model for Neighbor-Joining (NJ), the General Time Reversible (GTR) model with gamma distribution (+G), and the invariant sites (+I) model for Maximum Likelihood (ML) proposed by the corrected Akaike information criterion (AICc) in MEGA7 [42]. A *Xanthogramma citrofasciatum* (De Geer, 1776) sequence was included in the matrices as an outgroup. Then, collapsed matrices of unique haplotypes of COI-5′ (for *E. barbarus* and *E. sulcitibius*) and COI (for *E. gibbosus*) were performed with the software DNA Sequence Polymorphism (DnaSP) v6.12.03 [43]. The haplotype networks were constructed using the Median-Joining method in the software Population Analysis with Reticulate Trees (PopART) v1.7 [44] in order to shape the relationships between the specimens.

## 3. Results

### 3.1. Morphological Study

**Figure 1:**
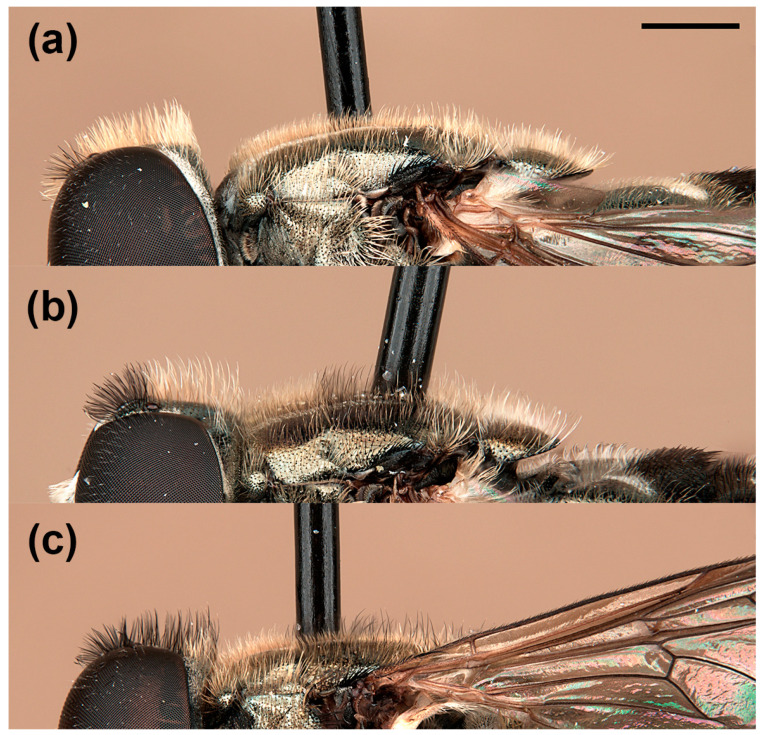
*Eumerus barbarus*, vertical triangle and scutum, pilosity (male): (**a**) mainly golden-yellow (but black on the ocellar triangle); (**b**) black on the vertical triangle anteriorly and intermixed on the scutum; (**c**) black on vertical triangle and intermixed on the scutum. Scale bar = 750 μm.

**Figure 2:**
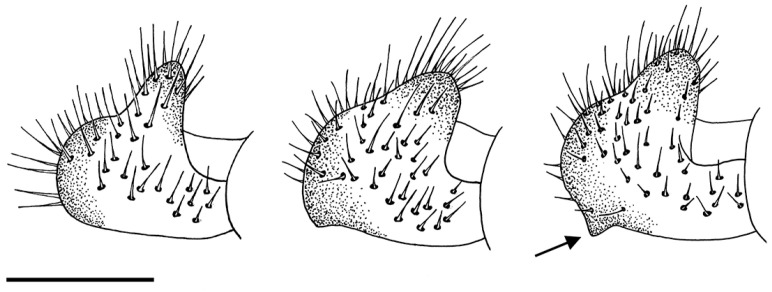
*Eumerus barbarus*, variation in the shape of the cercus of genitalia (male). An arrow indicates the triangular expansion. Scale bar = 250 μm.

**Figure 3:**
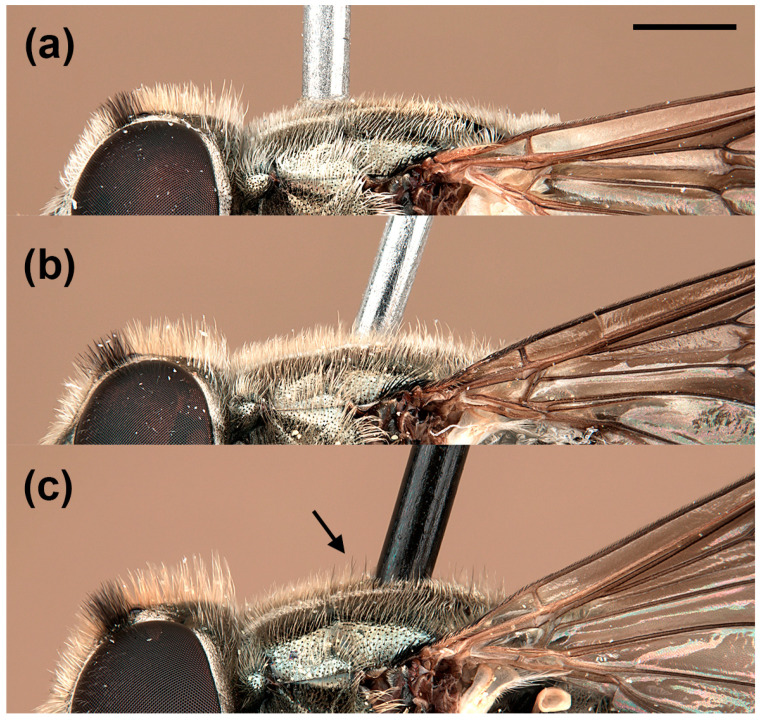
*Eumerus barbarus*, scutum, pilosity (female): (**a**) short yellow hairs; (**b**) long yellow hairs; (**c**) black and yellow hairs intermixed. An arrow indicates the black hairs. Scale bar = 1 mm.

**Figure 4:**
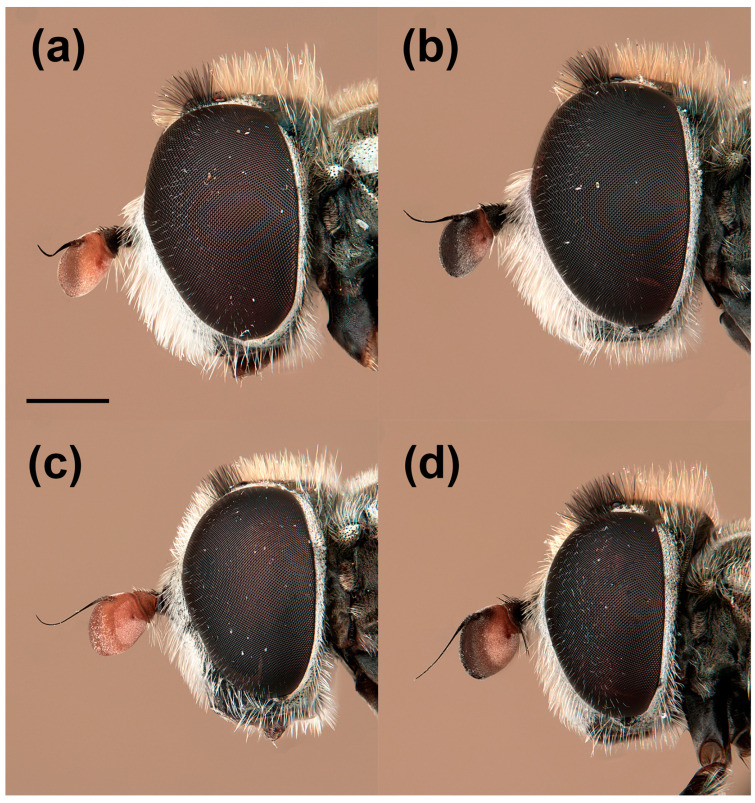
*Eumerus barbarus*, variation in the basoflagellomere coloration, male: (**a**) reddish orange, (**b**) blackish brown; female: (**c**) reddish orange, (**d**) blackish brown. Scale bar = 750 μm.

In addition to the variability reported by van Steenis et al. [5], males of *E. barbarus* displayed variation in the coloration of hairs on the posterior half of the vertical triangle, from golden-yellow to black; the arrangement of hairs on the scutum, from only golden-yellow hairs to long and black hairs intermixed with the yellow ones on the posterior half (Figure 1); and the shape of the cercus of the genitalia, from rounded distally to exhibiting a triangular expansion (Figure 2). The females of *E. barbarus* also revealed variation in the length of hairs on the scutum, from half the length of that on the vertical triangle to equal length; and in their coloration, from whitish yellow to black and yellow intermixed or completely black (Figure 3). Concerning the basoflagellomere ratio (length: width), previously studied by van Steenis et al. [5], we report new ratios for males (1.1:1.3 mm, *n* = 10) and females (0.9:1.2 mm, *n* = 10). We also illustrated the differentiation in the coloration of the basoflagellomere (Figure 4). In the case of males of *E. sulcitibius*, we report new ranges of body (6.2:8.5 mm, *n* = 10) and basoflagellomere (1:1.4 mm, *n* = 10) lengths. However, the most remarkable differentiation in males for this species was observed in the arrangement of hairs on the vertical triangle and mesonotum and the shape of male genitalia. The few studied specimens of *E. gibbosus* and *E. schmideggeri* revealed the same levels of variation as reported by van Steenis et al. [5].

### 3.2. Genetic Approach

**Figure 5:**
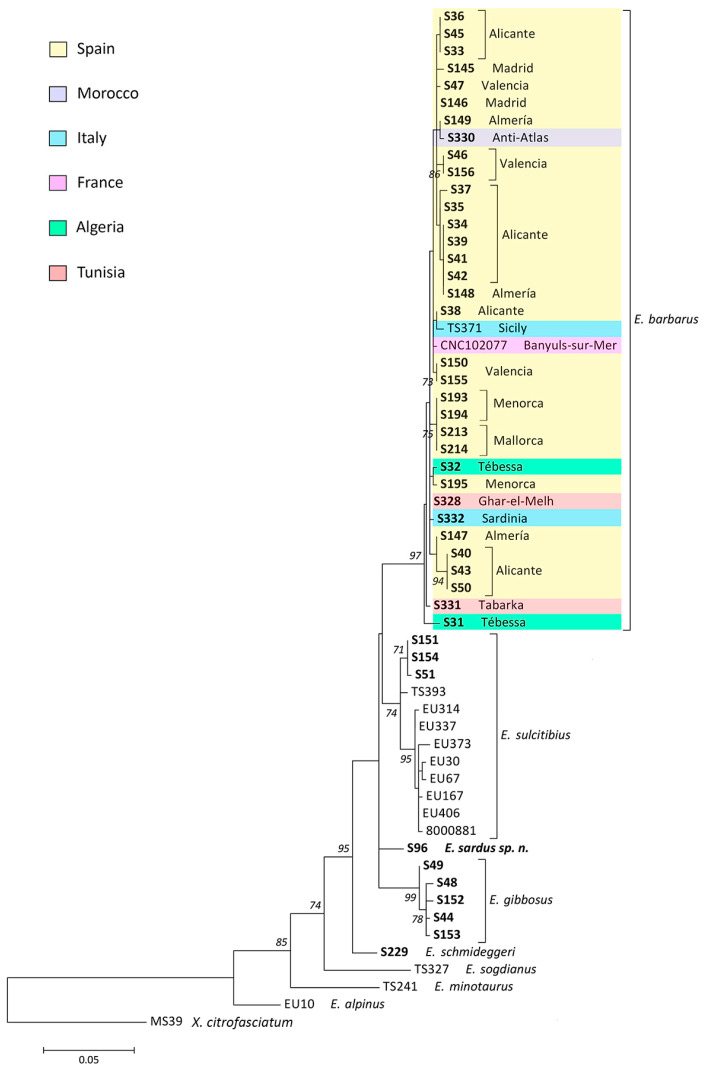
Maximum Likelihood tree based on COI-5′. We included only one sequence of each haplotype of *E. sulcitibius* from the eastern Mediterranean. DNA vouchers of specimens analyzed for this work are highlighted in bold. Bootstrap values >70 are shown near nodes. Branch lengths are measured in the number of substitutions per site.

**Figure 6:**
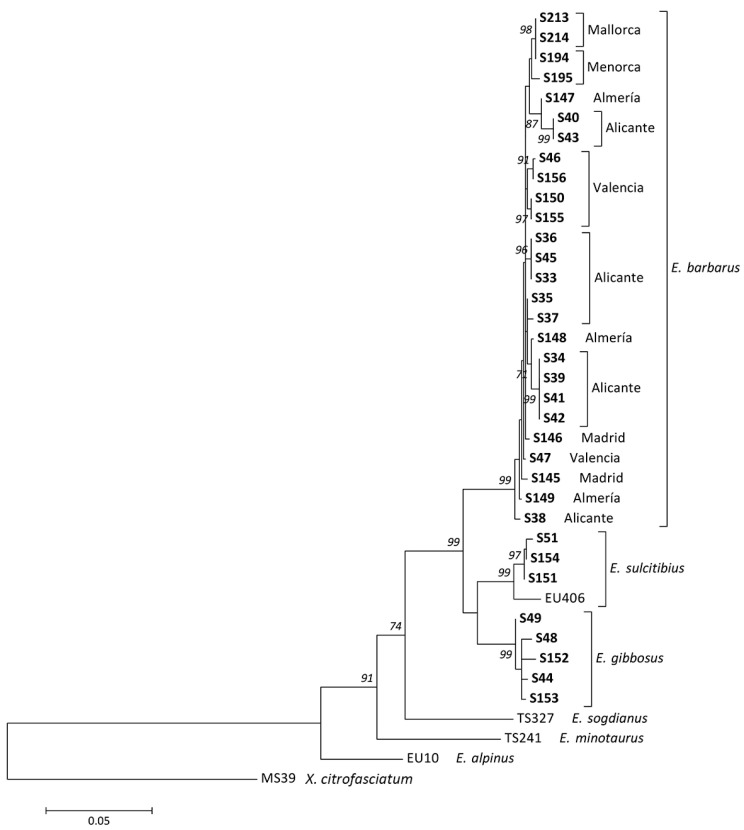
Maximum Likelihood tree based on COI (COI-5′+COI-3′). DNA vouchers of specimens analyzed for this work are highlighted in bold. Bootstrap values > 70 are shown near nodes. Branch lengths are measured in the number of substitutions per site.

**Figure 7:**
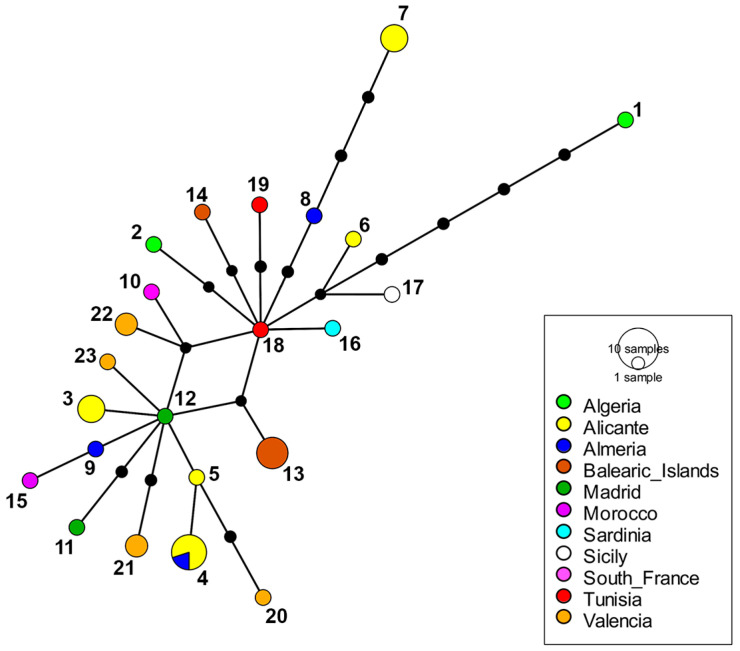
Haplotype network of *E. barbarus* from the western Mediterranean based on COI-5′ sequence data (see Supplementary Material S3). Size of circles is proportional to the number of individuals. Black dots depict the number of mutational steps. Three loops were removed in the network. Numbers of the haplotypes are indicated close to the circles.

**Figure 8:**
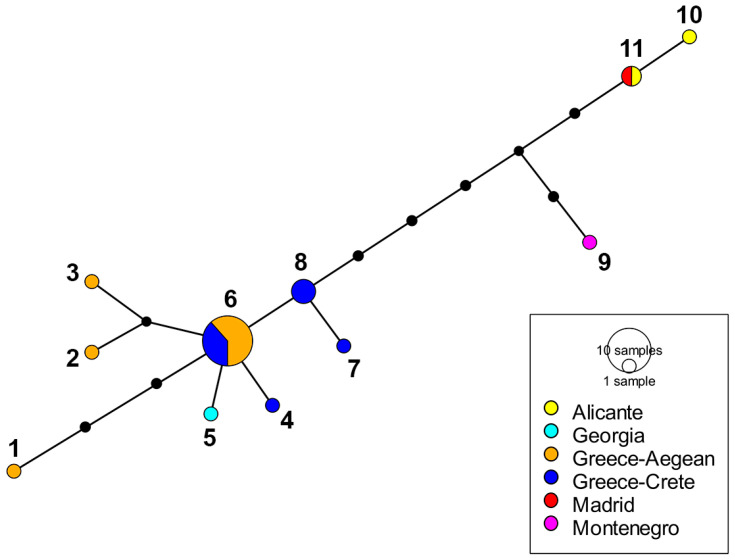
Haplotype network of *E. sulcitibius* from the Mediterranean area based on COI-5′ sequence data (see Supplementary Material S3). Size of circles is proportional to the number of individuals. Black dots depict the number of mutational steps. Numbers of the haplotypes are indicated close to the circles.

In the molecular study, COI (COI-5′+COI-3′) sequences of 26 specimens (14 males and 12 females) of *E. barbarus*, 5 specimens (2 males and 3 females) of *E. gibbosus* and 3 specimens (2 males and 1 female) of *E. sulcitibius* were generated. COI-5′ sequences were also obtained from 6 specimens of *E. barbarus* (5 males and 1 female), 1 male of *E. schmideggeri*, and 1 male of *Eumerus* aff. *sulcitibius* (see Supplementary Material S2). The resulting COI-5′–based tree clustered all species of the *E. barbarus* group in a well-supported clade (>90), displaying a polytomy (Figure 5). The COI-tree topology at species-group level was identical to that of the COI-5′-based tree (Figure 6). All analyzed specimens of *E. barbarus* clustered together in both trees. The specimens of *E. sulcitibius* also grouped together of which the analyzed specimen of *Eumerus* aff. *sulcitibius* appeared out as a single lineage (Figure 5). The analysis of COI-5′ haplotypes for *E. barbarus* revealed 23 haplotypes in the western Mediterranean, where 15 are only present in the Ibero-Balearic area (Figure 7). Regarding *E. sulcitibius*, two haplotypes were also found to be exclusive to the Iberian Peninsula (Figure 8). For *E. gibbosus*, five haplotypes in COI were disclosed (three from the province of Alicante, one from the province of Almería, and one from the province of Valencia), corresponding to each one of the analyzed specimens (see Supplementary Material S3).

### 3.3. New Species Description

**Figure 9:**
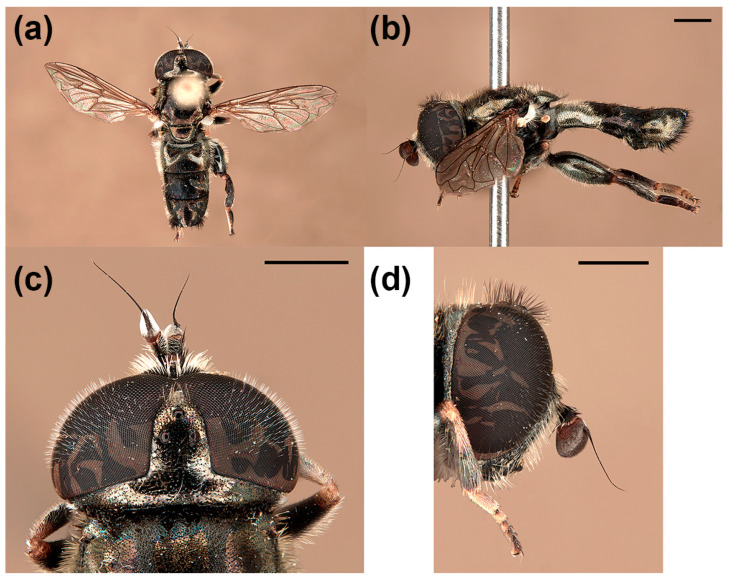
*Eumerus sardus* sp. n., holotype (male), habitus: (**a**) dorsal view, (**b**) lateral view; head: (**c**) dorsal view, (**d**) lateral view. Scale bars = (**a**,**b**) 1 mm; (**c**,**d**) 750 μm.

**Figure 10:**
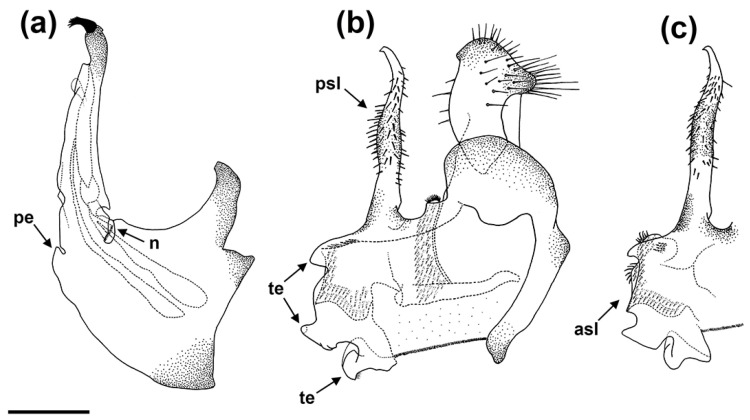
*Eumerus sardus* sp. n., holotype (male), genitalia: (**a**) hypandrium, lateral view; (**b**) epandrium, lateral view. *Eumerus sulcitibius*, male, genitalia: (**c**) surstylus, lateral view. Legend: asl, anterior surstylar lobe; n, notch; pe, pointed expansion; psl, posterior surstylar lobe; te, triangular expansion. Scale bar = 250 μm.

The examination of material from Sardinia, previously identified as *E. sulcitibius*, highlighted a great morphological disparity compared with the rest of the studied specimens of this species. After testing it through molecular data, a high level of dissimilarity in barcodes was revealed as well (Figure 1). Therefore, based on the combination of morphological and genetic evidence, the new species was characterized:

*Eumerus sardus* Aguado-Aranda, Ricarte & Hauser, sp. n. (Figure 9 and Figure 10)
=*Eumerus* aff. *sulcitibius*
urn:lsid:zoobank.org:act:FA53A8A6-D58C-4DE8-AF0C-1F31194385C4.

Holotype

ITALY • 1♂—I-Sardinien Lode, R. Mannu, April 1989, M. Hauser leg.//*Eumerus sulcitibius* Rond. {hand written}, det. Claußen 1989//*Eumerus sulcitibius* Rondani, 1868 ♂, det.: M. Hauser 1996 (CSCA).

Paratype

ITALY • 1♂—Italy, Sardinia, Monte Albo, 6 km SWW Siniscola, 40.558 N 9.634 E, 750 m, Schmid-Egger lg., 27.06.2017, l.sa12//DNA CEUA_S96//CEUA00113518 (CEUA-CIBIO).

Diagnosis. This species (only males, unknown female) can be distinguished from the other described species of the *E. barbarus* group by a posterior surstylar lobe slightly thickened medially and the general shape of the anterior surstylar lobe of the genitalia (Figure 10b).

Description. MALE (holotype). Body length = 7.44 mm. *Head.* Eyes contiguous. Eye hairs moderately long, covering the entire eye surface except the posterior area and the area close to the eye contiguity. Face and frontal triangle with slightly grey pollinose, covered with yellowish white hairs. Vertical triangle black, grey pollinose at its apex and along eye margin with two oval, grey pollinose maculae behind posterior ocelli; vertical triangle covered with long, erect black hairs which become golden-yellow toward the occiput; vertical triangle with yellow hairs at its apex. Ocellar triangle equilateral, with black hairs. Occiput black, grey pollinose; occiput with golden-yellow hairs which become shorter (than those on the vertical triangle) toward the laterals. Scape and pedicel black with long black and white hairs intermixed ventrally; pedicel light brown at its distal margin. Basoflagellomere oval (length:width ratio = 1.1:1); basoflagellomere predominately dark brown, darkish orange at its basal half. Pedicel and basoflagellomere sparsely white pollinose (pollinosity more obvious under artificial white lighting). Arista black and bare.

*Thorax*. Mesonotum and pleura black. Mesonotum with violet reflections (under artificial white lighting). Scutum with golden-yellow and black hairs intermixed, length of yellow hairs slightly surpassing half of the length of the black ones; scutum with three white pollinose vittae; lateral vittae slightly reaching 2/3 of the total length of the scutum; width of the lateral vittae equal to 1/8 of the total width of scutum; medial vitta less than 1/3 of the total length of the scutum; width of the medial vitta equal to 1/2 of the total width of one of the lateral vitta. Notopleural sulcus absent. Disc of scutellum with yellowish white and black hairs intermixed; length of that equal to the longest ones on the scutum (hairs on middle area of scutellum missing); posterior margin of the scutellum with small teeth-like protuberances, each bearing mainly a long black hair apically. Posterior anepisternum bearing long yellow hairs which become lighter ventrally but black hairs posteromedially. Katepisternum on its posterolateral area and anterior anepimeron and bearing densely arranged long white hairs. Katatergum with a discrete bunch of white hairs. Pleuron grey pollinose except the central area of the anterior anepimeron and the posterolateral margin of the katepisternum. Femora black, apices of the fore and mid femora light brown. Basal half of fore and mid tibiae light brown and distal half black. Hind tibia black, basal apex light brown. Fore and mid tarsomeres I–III brownish yellow; fore and mid tarsomeres IV–V dark brown. Hind tarsus dark brown dorsally and yellow ventrally. Fore and mid femora with long, backward directed, white hairs on their posterior sides. Hind coxa covered with long yellow hairs anteriorly; hind coxa posteriorly bare. Hind trochanter with a wing-like, flat, and backward directed expansion. Hind femur densely covered with yellow hairs. Hind femur with an anterior and posterior row of nine spinae each apicoventrally; hind femur with two strong, ventromedial spinae differentiated from the apicoventral rows of spinae. Hind femur with an apical bunch of short white hairs posteriorly. Hind tibia with a basoventral ridge covered with short, reclined, and black hairs; hind tibia with a broad lamina posteriorly; hind tibia with a basomedial concave depression. Wing membrane extensively microtrichose; posterior margin of the wing with dense, short, and brown hairs. Margin of ventral calypter with rather long and yellow hairs; hairs on the margin of dorsal calypter shorter than those on the ventral calypter; halter brownish yellow.

*Abdomen.* Terga I–IV black, with violet reflections (under artificial white lighting). Terga II–IV with a pair of slightly curved, white pollinose maculae. Middle area of terga II–IV covered with short, semi-reclined black hairs. Lateral margins of tergum I densely white pollinose. Tergum II with yellowish white hairs on lateral margins (hairs at the anterior corners rather long). Terga III–IV with short, white hairs on the lateral margins. Tergum IV with black hairs posteriorly. Sterna I–III dark brown; anterior margin of sternum I black. Sterna II–IV with yellowish white hairs. Sternum IV rectangular shaped and black, with yellow hairs; posterior margin of sternum IV v-shaped; sternum IV with a shallow, medial incision.

*Genitalia*. Epandrium with a posterior surstylar lobe elongated, slightly widened and curved medially, and with a pointed apex; posterior surstylar lobe densely covered with short black hairs (Figure 10b). Anterior surstylar lobe with three triangular expansions (Figure 10b). Cercus with long hairs. Interior accessory lobe densely covered with short hairs. Hypandrium simple, with a little, slightly pointed expansion basally and a concealed basodorsal notch (Figure 10a).

FEMALE. Unknown.

Etymology. This species is named after the Sardinians (also known as Sards), a Romance-language-speaking ethnic group native to Sardinia. The specific epithet “sardus” should be treated as an adjective in the nominative singular.

**Figure 11:**
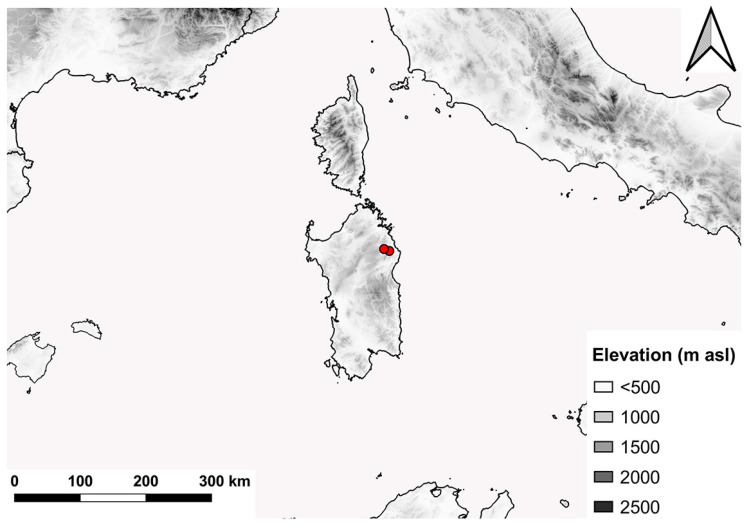
Distribution range of *E. sardus* sp. n.

Distribution. Northeast Sardinia (Figure 11).

Biology. The two adults were collected in April and June at lower altitudes (over 700 m asl).

### 3.4. Key to Males of the E. barbarus Group from the Western Mediterranean Basin

The key presented below is adapted from the key to the species of this group by van Steenis et al. [5].

1. Hind trochanter with a rounded-to-triangular, ventromedial expansion. Hind femur is thickened. Posterior surstylar lobe of male genitalia is slender and hook-shaped at the apex … *E. barbarus* group
  − A different combination of features … Other species groups

2. Hind femur with 2–3 long black spinae ventrally, clearly different from those of the apicoposterior row of spinae (van Steenis et al. [5]: figure 8d). Hind tibia with a concave depression medioventrally (van Steenis et al. [5]: figure 9b) … 3
  − Hind femur without those distinctive long spinae, only with the characteristic apicoventral rows of short spinae … 4

3. The entire surface of the scutum is covered with black and yellow hairs intermixed. Posterior surstylar lobe of genitalia is slightly thickened medially (Figure 10b) … *E. sardus* sp. n.
  − Anterior half of the scutum only with yellow hairs. Posterior surstylar lobe of genitalia is uniform in width (Figure 10c) … *E. sulcitibius*

4. Scutum covered with white hairs. Terga II–IV with broad, white pollinose maculae (van Steenis et al. [5]: figure 1f). Posterior surstylar lobe of the genitalia is rounded at its apex (van Steenis et al. [5]: figure 11c) … *E. schmideggeri*
  − Hairs on the scutum are predominately yellow (some black hairs may appear). Maculae on terga II–IV are narrow … 5

5. Hind femur remarkably thickened (van Steenis et al. [5]: figure 7a). Sternum IV with two posteromedial, rounded projections (van Steenis et al. [5]: figure 10a). Hypandrium without apicoventral expansions (van Steenis et al. [5]: figure 11a) … *E. barbarus*
  − Hind femur less thickened (van Steenis et al. [5]: figure 7b). Sternum IV is flat (van Steenis et al. [5]: figure 10b). Hypandrium with two, square-like expansions apicoventrally (van Steenis et al. [5]: figure 11b) … *E. gibbosus*

## 4. Discussion

### 4.1. Morphological Assessment

*Eumerus barbarus* displays high levels of morphological variability compared with the other species of the *E. barbarus* group. For example, males collected in the Alicante province showed golden-yellow hairs on the mesonotum but others from the same locality exhibited intermixed yellow and black hairs. Similarly, a black coloration of the hairs on the vertical triangle was observed mainly in specimens from the Balearic Islands compared with those from the mainland. The coloration of body hairs is highly variable in this species, but it represents a diagnostic character for other species of *Eumerus* (e.g., [45]). On the other hand, a variation in the shape of the cerci of male genitalia from different Iberian sites was also observed (Figure 2). This is not unusual because variation in genitalia within a species have also been reported for other *Eumerus*, namely, certain species of the *E. tricolor* group [16]. The phenotypic variation of *E. barbarus* was contrasted with the molecular data. Both COI-5′- and COI-based trees clustered all specimens in well-supported clades (>90) but displaying a polytomy (Figure 5 and Figure 6). This is not an isolated case as high phenotypic plasticity was reported in other syrphids; for instance, Ballester-Torres et al. [14] listed 16 variable features in more than 300 examined Iberian specimens of *Cheilosia ruffipes* (Preyssler, 1793) (as *Cheilosia soror* (Zetterstedt, 1843)). Contrary to our expectations, our results reflect that there is no cryptic species diversity in *E. barbarus* and corroborate taxonomic acts such as the synonymy of *Eumerus australis* Meigen, 1838, with *E. barbarus*. Otherwise, the integrative analyses revealed a hidden taxonomic diversity within *E. sulcitibus*. *Eumerus sardus* sp. n. differs from *E. sulcitibius* in terms of the hair coloration of the vertical triangle—mainly black on the anterior half in *E. sardus* sp. n. and yellow (but black on the ocellar triangle) in *E. sulcitibius*; the disposition of hairs on the scutum— black and yellow hairs intermixed over the entire surface in *E. sardus* sp. n. but on the posterior half in *E. sulcitibius*; hair coloration on the scutellum—black and golden-yellow intermixed in *E. sardus* sp. n. but mainly yellow in *E. sulcitibius*; the posterior surstylar lobe of male genitalia—slightly thickened medially in *E. sardus* sp. n. but uniform in width in *E. sulcitibius*; and the general shape of the anterior surstylar lobe of male genitalia (Figure 10b,c). *Eumerus sardus* sp. n. is the only known Sardinian-endemic hoverfly since the taxonomic status of *Merodon splendens* Hurkmans, 1993, is unclear (Ante Vujić, in litt.). After this study, the list of syrphid species recorded in Sardinia grows to 108 [21,46].

### 4.2. Spatial Genetic Diversity

The haplotype network evidenced 23 haplotypes of COI-5′ for *E. barbarus* in the western Mediterranean. Chroni et al. [17] observed similar values of variation in the Mediterranean populations of other species of *Eumerus*. Moreover, Mediterranean peninsulas acted as climate refuges during glaciations, which promoted genetic divergence and speciation events, which is reflected in the number of species and the amount of genetic diversity found in these areas [47]. The topography of southern Europe offered multiple shelters from climatic fluctuations, producing several genetic lineages within species [48]. Our results support this premise as 15 haplotypes of COI-5′ for *E. barbarus* were found in the Ibero-Balearic region (Figure 7). Despite the large geographical area considered in this work, genetic diversity of *E. barbarus* showed no spatial pattern, suggesting no genetically structured populations. A lack of a spatial genetic distribution might be a result of high connectivity between populations (i.e., gene flow) [22]. On the other hand, the levels of genetic diversity observed in the western Mediterranean populations of *E. barbarus* are consistent with the hypothesis that genetic divergence between populations of the same species increases at low latitudes (i.e., close to the Equator) [49]. In addition, the presence of different haplotypes on islands could be the result of the environmental conditions, which differ from those on the mainland [22]. For example, the uniqueness of the Balearic populations of *E. barbarus* may be indicative of evolutionary significant units [20]. The existence of evolutionary units on these islands is plausible, as has been shown in other taxonomic studies on the fauna of this archipelago (e.g., [50]). This fact highlights the importance of island ecosystems as diversification areas of fauna and flora. Although the existence of cryptic diversity (i.e., different taxa with very similar morphology) within *E. barbarus* is dismissed, the high genetic diversity of this species suggests an increased probability of speciation events in the future [49].

Regarding *E. sulcitibius*, the haplotype network appears to reflect a spatial distribution. Chroni et al. [17] reported two genetic clusters of this species in populations from the eastern Mediterranean. As in *E. barbarus*, two haplotypes of COI-5’ in *E. sulcitibius* are unique to the Iberian Peninsula (Figure 8). The finding of these haplotypes may suggest a third cluster in this region. However, as indicated by the latter authors, more exhaustive surveys are required in order to corroborate the structure of the Mediterranean populations of *E. sulcitibius*. Concerning *E. gibbosus*, the analysis of COI showed a star-like pattern of five haplotypes. Despite the small sample size (i.e., five adults), our results appear to reflect a high intraspecific genetic diversity in this species. Nevertheless, this should be taken with caution until a better geographical representation of this species at the molecular level is available.

### 4.3. Distribution and Conservation

**Figure 12:**
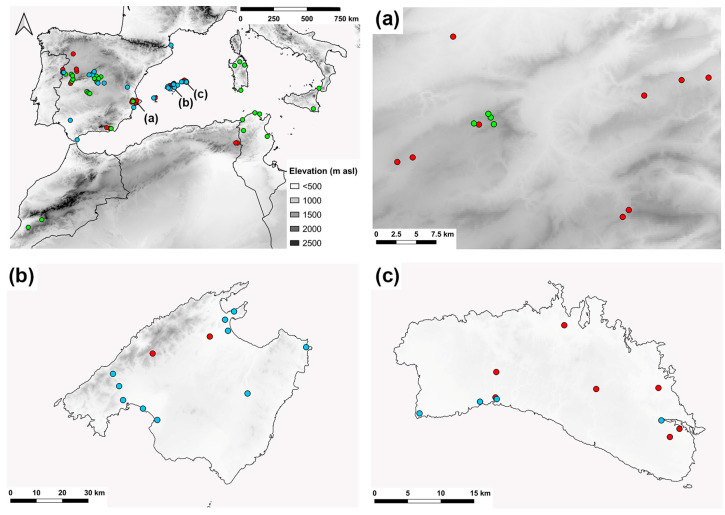
Distribution range of *E. barbarus* in the western Mediterranean basin. Magnifications: (**a**) Province of Alicante, (**b**) Mallorca, (**c**) Menorca. Red dots indicate new records, green dots indicate confirmed literature records, and blue dots indicate unconfirmed literature records.

**Figure 13:**
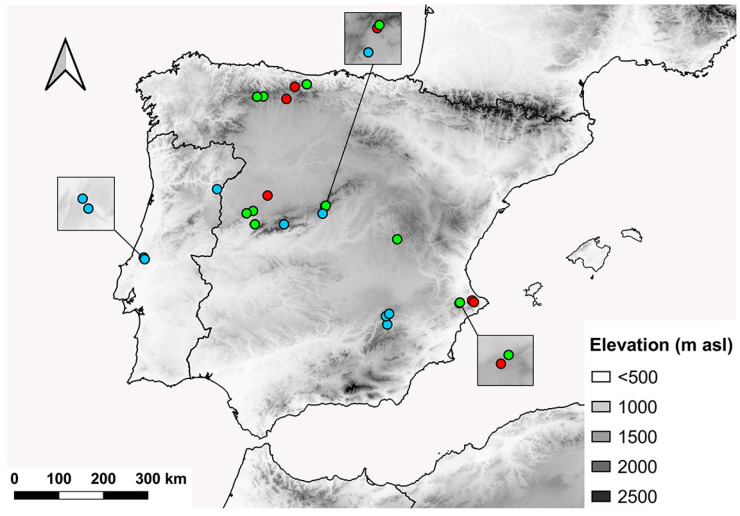
Distribution range of *E. sulcitibius* in the Ibero-Balearic area. Red dots indicate new records, green dots indicate confirmed literature records, and blue dots indicate unconfirmed literature records.

**Figure 14:**
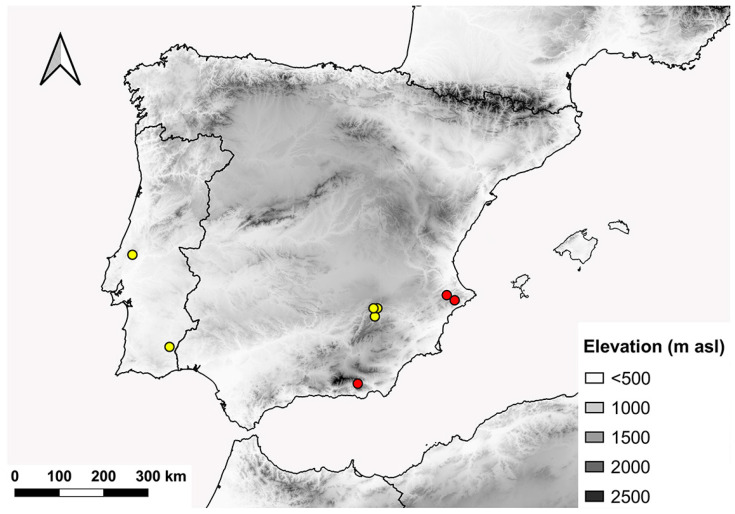
Distribution range of *E. gibbosus*. Red dots indicate new records and yellow dots indicate literature records.

**Figure 15:**
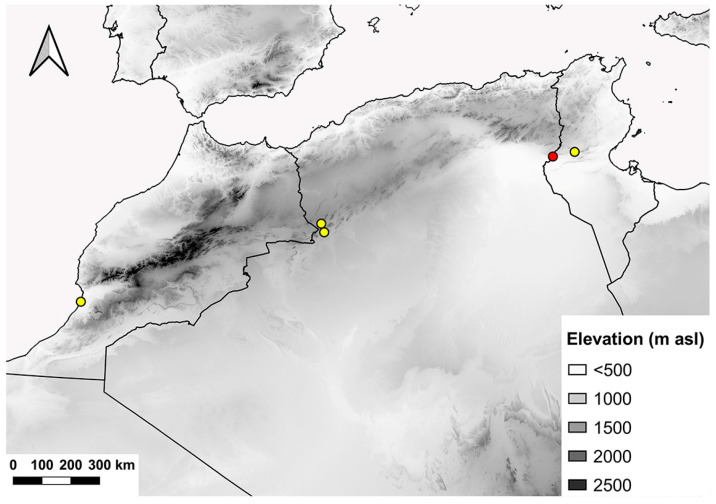
Distribution range of *E. schmideggeri*. Red dots indicate new records and yellow dots indicate literature records.

The Mediterranean basin hosts a high taxonomic diversity of hoverflies [51], but some genera, like *Eumerus* (e.g., [4,10]), are still understudied. The *E. barbarus* group is not an exception as other new species have been recently recorded in the Mediterranean area [52,53]. The species of the *E. barbarus* group have a mainly Mediterranean distribution and can be found in woodlands of *Quercus* L./*Pinus* L., open ground grasslands, xeric environments, and along rivers or seasonal water courses, among others [21]. Regarding the western Mediterranean, we reported new distribution records for the other studied species of the *E. barbarus* group, mainly from the Ibero-Balearic area (Figure 12, Figure 13, Figure 14 and Figure 15). For instance, *E. gibbosus* is recorded for the first time from the Spanish provinces of Alicante, Almería, and Valencia. The presence of geophytes is a key factor in the distribution ranges of *Eumerus* as early stages develop on the underground storage organs of these plants (e.g., [54,55]). For instance, preimaginal stages of *E. barbarus* appear to be reared from the bulbs of cultivated species of *Allium* L., but thus is pending confirmation [21]. So, no information about the feeding regimes (i.e., phytophagous or saprophagous) of the species of the *E. barbarus* group is available. This lack of information in the early stages has manifested in many published studies (e.g., [56,57]). According to the IUCN Red List categories and criteria [58], *E. barbarus* and *E. sulcitibius* are not threatened with extinction, whereas *E. gibbosus* is evaluated as endangered [59] and *E. schimideggeri* is still pending assessment. Therefore, future research on the life cycles and feeding regimes of these species is needed to shed light on the population trends of species of the *E. barbarus* group.

## Data Availability

All sequences generated in this work are available in the publicly accessible repository of GenBank (https://www.ncbi.nlm.nih.gov/genbank/).

## Supplementary Materials

The following supporting information can be downloaded at https://www.mdpi.com/article/10.3390/insects15040239/s1: S1: List of examined material; S2: List of species/specimens analyzed in the molecular study; S3: List of haplotypes.
