# Supplementary material for: Unveiling the Mainland vs. Insular Variability of the Eumerus barbarus Species Group (Diptera: Syrphidae) in the Western Mediterranean Basin
Source: Insects. 2024 Mar 29;15(4):239. doi: 10.3390/insects15040239 (PMC11050054; doi:10.3390/insects15040239)
Supplement: Supplementary file 1 [file insects-15-00239-s001.zip › Supplementary_material_S1_specimens.pdf]

Eumerus barbarus **(Coquebert, 1804)**

New. ALGERIA • 1♂ – Algérie, Tébessa, Cyprés, Mars 2017, *Eumerus strigatus*, Lég. Mebarkia Nadjoua // DNA CEUA_S31 // CEUA00108764 (CEUA-CIBIO) • 1♀ – Algérie, Tébessa, Pépinière, Juin 2017, *Eumerus strigatus*, Lég. Mebarkia Nadjoua // DNA CEUA_S32 / CEUA00108763 (CEUA-CIBIO) • 1♂ – Algérie, Tébessa, F Golden parc, 24 Mai 2012, *Eumerus barbarus*, Lég. Mebarkia Nadjoua, Det. S. Djellab // DNA CEUA_S376 // CEUA00113512 (CEUA-CIBIO) • SPAIN • 3♂♂ – ESPAÑA, Alicante, Alcoleja, Sierra Aitana, Puerto Tudons, campo de *Thapsia villosa*, 11-VI-2021, Leg.: Z. Nedeljković // CEUA00109930; 00109932; 00109938 (CEUA-CIBIO) • 3♀♀ – same data as for preceding // CEUA00110922; 00109950; 00109957 (CEUA-CIBIO) • 2♂♂ – same locality as for preceding, 06-VI-2020, Leg.: A. Ricarte // FAUNA IBERICA ERISTALINAE // DNA CEUA_S34 // CEUA00113519; 00107897 (CEUA-CIBIO) • 2♀♀ – same data as for preceding // FAUNA IBERICA ERISTALINAE // DNA CEUA_S42 // CEUA00107904; DNA CEUA_S45 // CEUA00107908 (CEUA-CIBIO) • 1♀ – same locality and date as for preceding, Leg.: Z. Nedeljković // FAUNA IBERICA ERISTALINAE // DNA CEUA_S50 // CEUA00107914 (CEUA-CIBIO) • 1♀ – same locality as for preceding, 29-V-2020, Leg.: A. Ricarte // FAUNA IBERICA ERISTALINAE // DNA CEUA_S40 // CEUA00107890 (CEUA-CIBIO) • 1♂ – same data as for preceding // FAUNA IBERICA ERISTALINAE // CEUA00107895 (CEUA-CIBIO) • 2♂♂ – same locality and date as for preceding, Leg.: Z. Nedeljković // FAUNA IBERICA ERISTALINAE // DNA CEUA_S38 // CEUA00107893; 00107896 (CEUA-CIBIO) • 1♀ – same data as for preceding // FAUNA IBERICA ERISTALINAE // CEUA00107901 (CEUA-CIBIO) • 2♂♂ – same locality and leg as for preceding, 26-V-2021 // CEUA00109931; 00109933 (CEUA-CIBIO) • 1♂ – same locality as for preceding, 19-VI-2020, Leg.: A. Ricarte // FAUNA IBERICA ERISTALINAE // DNA CEUA_S33 // CEUA00108760 (CEUA-CIBIO) • 1♂ – same locality and leg as for preceding, 20-VI-2020 // FAUNA IBERICA ERISTALINAE // DNA CEUA_S36 // CEUA00107898 (CEUA-CIBIO) • 1♀ – same locality and date as for preceding, Leg.: Z. Nedeljković // FAUNA IBERICA ERISTALINAE // CEUA00107903 (CEUA-CIBIO) • 2♀♀ – same locality as for preceding, 10-IX-2020, Leg.: A. Ricarte // DNA CEUA_S35 // CEUA00108762; 00107899 (CEUA-CIBIO) • 1♂ – same locality as for preceding, 18-IX-2021, Leg.: M. Hauser // CEUA00110929 (CEUA-CIBIO) • 1♀ – same locality as for preceding, 16-V-2021, Leg.: A. Ricarte // DNA CEUA_S41 // CEUA00107902 (CEUA-CIBIO) • 1♀ – same locality and date as for preceding, Leg.: Z. Nedeljković // CEUA00107900 (CEUA-CIBIO) • 1♀ – same locality as for preceding, 01-X-2020, Leg.: P. Aguado // DNA CEUA_S39 // CEUA00107891 (CEUA-CIBIO) • 2♀♀ – ESPAÑA, Alicante, Alcoleja, Sierra de Aitana, Mas del Piscul, 29-V-2019, Leg.: Antonio Ricarte // CEUA00107892; 00107905 (CEUA-CIBIO) • 1♀ – same locality and date as for preceding, Leg.: Zorica Nedeljković // DNA CEUA_S43 // CEUA00107906 (CEUA-CIBIO) • 1♀ – ESPAÑA, Alicante, Alcoleja, Sierra de Aitana, Mas del Piscul, en campos de cereal, 1-V-2019, Leg.: Antonio Ricarte // CEUA00107907 (CEUA-CIBIO) • 3♀♀ – ESPAÑA, Alicante, Alcoleja, Sierra Aitana, Mas del Piscual, campo de *Thapsia villosa*, 26-V-2021, Leg.: I. Ballester // CEUA00109946; 00110924; 00110928 (CEUA-CIBIO) • 1♀ – ESPAÑA (A), la Vall d'Alcalà, 38º48′10.05′′N 0º14′2.97′′O, 626m, campo *Thapsia villosa*, 20-V-2021, Leg.: Z. Nedeljković // CEUA00107929 (CEUA-CIBIO) • 1♀ – ESPAÑA, Alicante, la Vall d'Ebo, 38º48′16.47′′N 0º11′42.97′′O, 546m, campo *Thapsia villosa*, 20-V-2021, Leg.: Z. Nedeljković // CEUA00107912 (CEUA-CIBIO) • 1♂ – ESPAÑA, Alicante, la Vall d'Ebo, 38º48′16.60′′N 0º11′59.57′′O, 555m, 26-V-2021, Leg.: P. Aguado // CEUA00109927 (CEUA-CIBIO) • 1♀ – same locality and date as for preceding, Leg.: A. Ricarte / CEUA00109947 (CEUA-CIBIO) • 1♀ – ESPAÑA, Alicante, Planes, 38º47′11.68′′N 0º17′19.25′′O, 567m, campo *Thapsia villosa*, 20-V-2021, Leg.: Z. Nedeljković // CEUA00107911 (CEUA-CIBIO) • 1♀ – same locality and date as for preceding, Leg.: A. Ricarte // CEUA00107910 (CEUA-CIBIO) • 3♂♂ – ESPAÑA, Alicante, Banyeres de Mariola, La Mallaeta, 26-V-2021, en *Thapsia villosa*, Leg.: I. Ballester-Torres // CEUA00109926; 00109935-36 (CEUA-CIBIO) • 1♀ – same data as for preceding // CEUA00109948 (CEUA-CIBIO) • 13♂♂ – ESPAÑA, Almería, Sª de Gádor, Huécija, Camino Cerro de la Cruz, camino a la Ermita, 25-V-2022, Leg.: P. Aguado // CEUA00113572-84 (CEUA-CIBIO) • 2♀♀ – same data as for preceding // CEUA00113585-86 (CEUA-CIBIO) • 10♂♂ – same locality and data as for preceding, Leg.: Z. Nedeljković // CEUA00113587-96 (CEUA-CIBIO) • 2♀♀ – same data as for preceding // CEUA00113597-98 (CEUA-CIBIO) • 4♂♂ – same locality and date as for

preceding, Leg.: I. Ballester // CEUA00113599-03 (CEUA-CIBIO) • 4♀♀ – same data as for preceding // CEUA00113604-07 (CEUA-CIBIO) • 2♂♂ – ESPAÑA, Almería, Fondón, Bajo Viaducto Río Andarax, 36º59´13´´N 2º50´39´´W, 4-X-2021, Leg.: P. Aguado-Aranda // CEUA00110945; 00110947 (CEUA-CIBIO) • 4♂♂ – same locality and leg as for preceding, 5-X-2021 // DNA CEUA_S148 // CEUA00110946; DNA CEUA_S147 // CEUA00110949; 00110950-51 (CEUA-CIBIO) • 1♀ – same data as for preceding // DNA CEUA_S149 // CEUA00110948 (CEUA-CIBIO) • 2♂♂ – ESPAÑA (IB), Mallorca, Sa Pobla, campo de cultivo, en *Daucus* sp., 16-VI-2022, Leg.: A. Ricarte // CEUA00113453-54 (CEUA-CIBIO) • 1♂ – ESPAÑA (IB), Mallorca, Bunyola, Orient, hotel Nou Dalt Muntanya, 15-VI-2022, Leg.: Z. Nedeljković // DNA CEUA_S213 // CEUA00113451 (CEUA-CIBIO) • 1♂ – same locality and date as for preceding, Leg.: A. Ricarte // DNA CEUA_S214 // CEUA00113452 (CEUA-CIBIO) • 5♂♂ – España (IB), Menorca, Maó, Poblat Talaiotic de Trepucó, 39.87 4.265, 60 m, 13-V-2022, Leg.: A. Ricarte & Z. Nedeljković // En flores de *Daucus* sp. // DNA CEUA_S194 // CEUA00111034; 00111036-39 (CEUA-CIBIO) • 1♀ – same data as for preceding // En flores de *Daucus* sp. // DNA CEUA_S195 // CEUA00111035 (CEUA-CIBIO) • 4♂♂ – España (IB), Menorca, Es Castell, cerca Me-2, 39.88 4.283, 31 m, 13-V-2022, Leg.: A. Ricarte & Z. Nedeljković // DNA CEUA_S193 // CEUA00111030-32; 00111041 (CEUA-CIBIO) • 2♀♀ – same data as for preceding // CEUA00111033; 00111040 (CEUA-CIBIO) • 1♂ – España (IB), Menorca, Es Mercadal, Prat de Tirant, 40.033 4.095, 2 m, 15-V-2022, Leg.: P. Aguado // CEUA00111042 (CEUA-CIBIO) • 2♀♀ – same data as for preceding // CEUA00111043-44 (CEUA-CIBIO) • 2♂♂ – same locality and date as for preceding, Leg.: A. Ricarte // CEUA00111045-46 (CEUA-CIBIO) • 1♀ – España (IB), Menorca, Barranc d'Algendar, 15-V-2022, Leg.: Z. Nedeljković // CEUA00111047 (CEUA-CIBIO) • 16♂♂ – España, Menorca, s'Albufera des Grau, aparcamiento, 29-IV-2023, 20 m, 39º56´16´´N 4º15´7´´E, Leg. P. Aguado-Aranda // CEUA00114664-78; 00114680 (CEUA-CIBIO) • 7♀♀ – same data as for preceding // CEUA00114681-87 (CEUA-CIBIO) • 2♂♂ – same locality and date as for preceding, Leg. I. Ballester-Torres // CEUA00114679; 00114688 (CEUA-CIBIO) • 3♀♀ – same data as for preceding // CEUA00114689-90; 00115193 (CEUA-CIBIO) • 8♂♂ – same locality and date as for preceding, Leg. Z. Nedeljković // CEUA00114691-96; 00114698-99 (CEUA-CIBIO) • 7♀♀ – same data as for preceding // CEUA00114700-07 (CEUA-CIBIO) • 1♂ – España, Menorca, Cala Galdana, laterales del río, 02-V-2023, Leg. Z. Nedeljković // CEUA00114697 (CEUA-CIBIO) • 3♂♂ – España, Menorca, Poblado Talayótico de Trepucó, 29-IV-2023, Leg. Aguado, Ballester, Nedeljković // CEUA00114708-10 (CEUA-CIBIO) • 2♀♀ – same data as for preceding // CEUA00114711-12 (CEUA-CIBIO) • 2♂♂ – España, Menorca, Barranc d'Algendar, 30-IV-2023, 30 m, 39º58´37´´N 3º58´7´´E, Leg. Z. Nedeljković // CEUA00114713-14 (CEUA-CIBIO) • 1♂ – same locality and date as for preceding, Leg. I. Ballester-Torres // CEUA00114715 (CEUA-CIBIO) • 2♂♂ – same locality and date as for preceding, Leg. P. Aguado-Aranda // CEUA00114716-17 (CEUA-CIBIO) • 4♀♀ – same data as for preceding // CEUA00114718-19; 00115183-84 (CEUA-CIBIO) • 8♂♂ – same locality and leg as for preceding, 3-X-2023 // CEUA00113530-37 (CEUA-CIBIO) • 3♂♂ – same locality and date as for preceding, Leg.: Z. Nedeljković & I. Ballester // CEUA00113538-40 (CEUA-CIBIO) • 1♀ – same data as for preceding // CEUA00113541 (CEUA-CIBIO) • 3♂♂ – España (IB), Menorca, Alaior, cerca de cementerio, en *Phoeniculum vulgare*, 1-X-2023, Leg. Z. Nedeljković // CEUA00113520-22 (CEUA-CIBIO) • 3♂♂ – same locality and date as for preceding, Leg.: P. Aguado Aranda // CEUA00113523-25 (CEUA-CIBIO) • 1♀ – same locality and date as for preceding, Leg.: I. Ballester Torres / CEUA00113526 (CEUA-CIBIO) • 1♂ – same data as for preceding // CEUA00113527 (CEUA-CIBIO) • 2♂♂ – same locality as for preceding, 13-VII-2023. Leg.: Z. Nedeljković & M.Á. Marcos // CEUA00113528-29 (CEUA-CIBIO) • 1♂ – San Agustín (IBIZA), 12-III-1997, Leg.: Marcos-García & Rojo // *Eumerus barbarus* (Coquebert, 1804), Det.: A. Ricarte, 2005 // CEUA00017777 (CEUA-CIBIO) • 1♂ – Puigden Trias (IBIZA), 12-III-1997, Leg.: Marcos-García & Rojo // *Eumerus barbarus* (Coquebert, 1804), Det.: A. Ricarte, 2005 // CEUA00017776 (CEUA-CIBIO) • 2♂♂ – ESPAÑA, Madrid, Cercedilla, borde Ctra. M-622, 40º44´10´´N 4º2´15.7´´O, 1200 m, 27-VII-2021, Leg.: A. Ricarte // En flores de *Magydaris panacifolia* // DNA CEUA_S145 // CEUA00109928; DNA CEUA_S146 // CEUA00109939 (CEUA-CIBIO) • 1♂ – ESPAÑA, Valencia, Alt del Portell, Bocairent, Vall 'Albaida, 837m, 12-VI-2021, Leg.: A. Ricarte // CEUA00109929 (CEUA-CIBIO) • 9♀♀ – same data as for preceding // CEUA00109944; 00109949; 00109951-54;

00109956; 00109958-59; 00110925-26 (CEUA-CIBIO) • 1♂ – same locality and data as for preceding, Leg.: Z. Nedeljković // CEUA00109937 (CEUA-CIBIO) • 8♀♀ – same data as for preceding // CEUA00109941-43, 00109945; 00109955; 00110920-21; 00110927 (CEUA-CIBIO) • 2♀♀ – ESPAÑA, Valencia, Sª Mariola, Bocairent, Font del Mas dels Arbres, campo de *Thapsia villosa*, 03-VI-2020, Leg.: Z. Nedeljković // FAUNA IBERICA ERISTALINAE // DNA CEUA_S47 // CEUA00107918; DNA CEUA_S46 // CEUA00107919 (CEUA-CIBIO) • 1♂ – same locality and date as for preceding, Leg.: A. Ricarte // FAUNA IBERICA ERISTALINAE // DNA CEUA_S37 // CEUA00107894 (CEUA-CIBIO) • 1♂ – same locality as for preceding, 29-V-2021, Leg.: I. Ballester // CEUA00109934 (CEUA-CIBIO) • 1♀ – same data as for preceding // CEUA00110923 (CEUA-CIBIO) • 5♂♂ – ESPAÑA, Valencia, Bocairent, Font dels Brulls, 22-VII-2021, 775m asl, Leg.: I. Ballester-Torres // DNA CEUA_S155 // CEUA00110953; DNA CEUA_S156 // CEUA00110957; 00110955; 00110958-59 (CEUA-CIBIO) • 2♀♀ – same data as for preceding // DNA_S150 // CEUA00110956; 00110954 (CEUA-CIBIO) • 1♀ – Sitrama de Tera (ZAMORA), 4-VIII-1986, Leg.: Mª.A. Marcos {hand written} // SYRPHIDAE, *Eumerus barbarus* (Coquebert), Det.: Mª. A. Marcos-García // CEUA00017778 (CEUA-CIBIO) • 1♂ – Malpartida de Plasencia (CC), 500 m, 7-VI-80, Leg.: Mª.A. Marcos {hand written} // SYRPHIDAE, *Eumerus barbarus* (Coquebert), Det.: Mª. A. Marcos-García // CEUA00017771 (CEUA-CIBIO) • 1♀ – El Torno (CC), 950 m, 25-VIII-80, Leg. Mª.A. Marcos {hand written} // SYRPHIDAE, *Eumerus barbarus* (Coquebert), Det.: Mª. A. Marcos-García // CEUA00017780 (CEUA-CIBIO) • 1♀ – La Flecha-Cabrerizos, 7-IX-78, Leg. Mª.A. Marcos // SYRPHIDAE, *Eumerus barbarus* (Coquebert), Det.: Mª. A. Marcos-García // CEUA00017779 (CEUA-CIBIO) • 1♀ – Alba de Tormes (SA), 2-VII-79, Leg. Mª.A. Marcos {hand written} // SYRPHIDAE, *Eumerus barbarus* (Coquebert), Det.: Mª. A. Marcos-García // CEUA00017784 (CEUA-CIBIO) • 1♂ – Arribes del Duero (SA), 13-V-88, Leg.: Mª.A. Marcos {hand written} // SYRPHIDAE, *Eumerus barbarus* (Coquebert), Det.: Mª. A. Marcos-García // CEUA00017773 (CEUA-CIBIO).

Revised. ITALY • 1♂ – Messina, 53788.IV {hand written} // *Eumerus australis* {hand written} // Frank M. Hull Collection C.N.C. 1973 // CNC DIPTERA # 155809 (CNC) • 1♂ – RAGUSA, Italia, 8-5-1956, F.F. Tippmann {leg} // *Eumerus barbarus* Coq. ♂, det. v. Doesburg // CNC DIPTERA # 155827 (CNC) • 1♂ – St. Margherita, SARDINIA, 29.V.1965, J.W. Boyes // SS 14 // *Eumerus barbarus* Coq. ♂, det. v. Doesburg // CNC DIPTERA # 155826 (CNC) • 1♂ – I-Sardinien, April 1989, Florinas, 417m, M. Hauser leg. // *Eumerus barbarus* Coqu. {hand written}, det. Claußen 1989 // *Eumerus barbarus* (Coquebert, 1804) ♂, det.: M. Hauser 1996 // DNA CEUA_S332 (CSCA) {published in van Steenis et al. [5]} • 1♀ – I-Sardinien, Tempio, 1200m, Mt. Limbara, 20.7.96, Leg.: Dr. Ch. L. Neumann // DNA CEUA_S333 (CSCA) {published in van Steenis et al. [5]} • 1♀ – I-Sardinien, Lode, R. Mannu, April 1989, M. Hauser leg. // *Eumerus barbarus* (Coquebert, 1804) ♀, det.: M. Hauser 1996 (CSCA) {published in van Steenis et al. [5]} • 1♂ – Messina, 53788. IV. {no further details} // *barbarus* Coqueb. // MNCN_Ent 142879 // SYRPHIDAE, Eristalinae, *Eumerus barbarus* (Coquebert, 1804), Det.: P. Aguado-Aranda, 2021 (MNCN) • MOROCCO • 2♂♂ – Marokko, Antiatlas Ammelental, 10 km NE Tafraoute, 14.III.97, 29º48´N 8º53´W, Leg. M. Hauser // DNA CEUA_S330 (CSCA) {published in van Steenis et al. [5]} • 1♂ – Marokko, Antiatlas, S Ait-Baha, 12.III.97, 30º00´N 9º02´W, Leg. M. Hauser (CSCA) {published in van Steenis et al. [5]} • 1♂ – Marokko, 11km NW Taliouine, 15.III.97, 30º34´N 8º00´W, Leg. M. Hauser (CSCA) {published in van Steenis et al. [5]} • SPAIN • 1♂ – Spain, Almería province, 800 ft, 3 km W Benahadux, 11-IV-1999, ME Irwin, 36º55.11´N 02º28.64´W, hand netted (CSCA) {published in van Steenis et al. [5]} • 1♂ – El Torno (CC), 400 m, 17-V-80, Leg. Mª.A. Marcos {hand written} // SYRPHIDAE, *Eumerus barbarus* (Coquebert), Det.: Mª. A. Marcos-García // CEUA00017772 (CEUA-CIBIO) {published in Marcos-García [27]} • 1♂ – El Torno (CC), 350 m, 4-IV-81, Leg. Mª.A. Marcos {hand written} // SYRPHIDAE, *Eumerus barbarus* (Coquebert), Det.: Mª. A. Marcos-García // CEUA00017774 (CEUA-CIBIO) {published in Marcos-García [27]} • 1♀ – Rinconada de la Sierra (SA), 17-VI-80, Leg.: Mª.A. Marcos {hand written} // SYRPHIDAE, *Eumerus barbarus* (Coquebert), Det.: Mª. A. Marcos-García // CEUA00017783 (CEUA-CIBIO) {published in Marcos-García [27]} • 1♀ – S. Esteban de la Sierra (SA), 2-VII-80, Leg.: Mª.A. Marcos {hand written} // SYRPHIDAE, *Eumerus barbarus* (Coquebert), Det.: Mª. A. Marcos-García // CEUA00017782 (CEUA-CIBIO) {published in Marcos-García [27]} • 1♀ – Hervás (CC), 600 m, 28-X-80, Leg.: Mª.A. Marcos {hand written} //

SYRPHIDAE, *Eumerus barbarus* (Coquebert), Det.: Mª. A. Marcos-García // CEUA00017781 (CEUA-CIBIO) {published in Marcos-García [27]} • 2♂♂ – España, Ciudad Real, P.N. de Cabañeros, maR1 {Malaise trap}, 1/24-VIII-2004, Leg.: A. Ricarte // SYRPHIDAE, *Eumerus barbarus* (Coquebert, 1804) ♂, Det.: A. Ricarte 2006 // CEUA00083342; 00083344 (CEUA-CIBIO) {published in Ricarte [30]} • 1♀ – same data as for preceding // SYRPHIDAE, *Eumerus barbarus* (Coquebert, 1804) ♀, Det.: A. Ricarte 2006 // CEUA00083351 (CEUA-CIBIO) {published in Ricarte [30]} • 1♀ – same locality, date and leg as for preceding, maR2 {Malaise trap} // SYRPHIDAE, *Eumerus barbarus* (Coquebert, 1804) ♀, Det.: A. Ricarte 2006 // CEUA00083352 (CEUA-CIBIO) {published in Ricarte [30]} • 1♀ – same locality and leg as for preceding, maR1 {Malaise trap}, 24-VIII/12-IX-2004 // SYRPHIDAE, *Eumerus barbarus* (Coquebert, 1804) ♀, Det.: A. Ricarte 2006 // CEUA00083349 (CEUA-CIBIO) {published in Ricarte [30]} • 3♀♀ – same locality, Malaise trap and leg as for preceding, 12-IX/08-X-2004 // SYRPHIDAE, *Eumerus barbarus* (Coquebert, 1804) ♀, Det.: A. Ricarte 2006 // CEUA00083347-49 (CEUA-CIBIO) {published in Ricarte [30]} • 1♀ – same locality and leg as for preceding, maM1 {Malaise trap}, 14-IV/8-V-2004 // *Eumerus barbarus* (Coquebert, 1804), Det.: A. Ricarte, 2005 // CEUA00083346 (CEUA-CIBIO) {published in Ricarte [30]} • 1♀ – same locality and leg as for preceding, maF2 {Malaise trap}, 11-IX/7-X-2004 // SYRPHIDAE, *Eumerus barbarus* (Coquebert, 1804) ♀, Det.: A. Ricarte 2006 // CEUA00083350 (CEUA-CIBIO) {published in Ricarte [30]} • 1♀ – same locality and leg as for preceding, Ap2 {sampling site}, 5-VII-2004 // *Eumerus barbarus* (Coquebert, 1804), Det.: A. Ricarte, 2005 // CEUA00083345 (CEUA-CIBIO) {published in Ricarte [30]} • 1♀ – Mas del Parral, 900 m, Bocairent (VALENCIA), 2-16/VII/02 {Malaise trap}, Leg.: Pérez-Bañón, Marcos-García & Rojo // CEUA00110944 (CEUA-CIBIO) {published in Mengual [29]} • 1♀ – same locality and leg as for preceding, 14-30/VIII/01 {Malaise trap} // *Eumerus barbarus* (Coquebert, 1804), Det.: A. Ricarte, 2005 // CEUA00110962 (CEUA-CIBIO) {published in Mengual [29]} • 1♀ – same locality and leg as for preceding, 31/VII-14/VIII/01 {Malaise trap} // *Eumerus barbarus* (Coquebert, 1804), Det.: A. Ricarte, 2005 // CEUA00110968 (CEUA-CIBIO) {published in Mengual [29]} • 2♂♂ – same locality and leg as for preceding, 30/VII-13/VIII/02{Malaise trap} // CEUA00110964; 00110970 (CEUA-CIBIO) {published in Mengual [29]} • 1♀ – same data as for preceding // *Eumerus barbarus* (Coquebert, 1804), Det.: A. Ricarte, 2005 // CEUA00110980 (CEUA-CIBIO) {published in Mengual [29]} • 1♀ – same locality and leg as for preceding, 3-17/VII/01 {Malaise trap} // *Eumerus barbarus* (Coquebert, 1804), Det.: A. Ricarte, 2005 // CEUA00110981 (CEUA-CIBIO) {published in Mengual [29]} • 1♀ – same locality and leg as for preceding, 16-30/VII/02 {Malaise trap} // *Eumerus barbarus* (Coquebert, 1804), Det.: A. Ricarte, 2005 // CEUA00110965 (CEUA-CIBIO) {published in Mengual [29]} • 1♂ – same data as for preceding // CEUA00110969 (CEUA-CIBIO) {published in Mengual [29]} • 1♀ – same locality and leg as for preceding, 24/IX-7/X/02 {Malaise trap} // *Eumerus barbarus* (Coquebert, 1804), Det.: A. Ricarte, 2005 // CEUA00110966 (CEUA-CIBIO) {published in Mengual [29]} • 1♂ – Foia Ampla, 1060 m, Agres (ALICANTE), 10-23/IV/02 {Malaise trap}, Leg.: Pérez-Bañón, Marcos-García & Rojo // *Eumerus barbarus* (Coquebert, 1804), Det.: A. Ricarte, 2005 / CEUA00110971 (CEUA-CIBIO) {published in Mengual [29]} • 1♂ – same locality and leg as for preceding, 30/VII-13/VIII/02 {Malaise trap} // CEUA00110967 (CEUA-CIBIO) • 1♀ – Caveta del Voltor, 1200 m, Agres (ALICANTE), 15/V-4/VI/02 {Malaise trap}, Leg.: Pérez-Bañón, Marcos-García & Rojo // *Eumerus barbarus* (Coquebert, 1804), Det.: A. Ricarte, 2005 // CEUA00110963 (CEUA-CIBIO) {published in Mengual [29]} • 1♀ – Font Retura, 900 m, Agres (Alicante), 30/VIII-2/IX/01 {Malaise trap}, Leg.: Pérez-Bañón, Marcos-García & Rojo // *Eumerus barbarus* (Coquebert, 1804), Det.: A. Ricarte, 2005 // CEUA00113511 (CEUA-CIBIO) {published in Mengual [29]} • 1♀ – Caveta del Voltor, 1200 m, Agres (ALICANTE), 15/V-4/VI-02 {Malaise trap}, Leg.: Pérez-Bañón, Marcos-García & Rojo // *Eumerus barbarus* (Coquebert, 1804), Det.: A. Ricarte, 2005 // CEUA00110974 (CEUA-CIBIO) {published in Mengual [29]} • 3♀♀ – same locality and leg as for preceding, 4-17/VI/02 {Malaise trap} // *Eumerus barbarus* (Coquebert, 1804), Det.: A. Ricarte, 2005 // CEUA00110975-77 (CEUA-CIBIO) {published in Mengual [29]} • 1♀ – same locality and leg as for preceding, 19/VI-3/VII/01 {Malaise trap} // *Eumerus barbarus* (Coquebert, 1804), Det.: A. Ricarte, 2005 // CEUA00110972 (CEUA-CIBIO) {published in Mengual [29]} • 1♀ – same locality and leg as for preceding, 27/VIII-10/IX/02 {Malaise trap} // *Eumerus barbarus* (Coquebert, 1804), Det.: A. Ricarte, 2005 // CEUA00110973 (CEUA-CIBIO) {published in Mengual [29]} • 2♀♀ – same locality

and leg as for preceding, 5-19/VI/01 {Malaise trap} // *Eumerus barbarus* (Coquebert, 1804), Det.: A. Ricarte, 2005 // CEUA00110979; 00110982 (CEUA-CIBIO) {published in Mengual [29]} • 1♀ – same locality and leg as for preceding, 23/V-5/VI/01 {Malaise trap} // *Eumerus barbarus* (Coquebert, 1804), Det.: A. Ricarte, 2005 // CEUA00110978 (CEUA-CIBIO) {published in Mengual [29]} • 1♂ – Seseña {Toledo} // MNCN_Ent 142913 // SYRPHIDAE, Eristalinae, *Eumerus barbarus* (Coquebert, 1804), Det.: P. Aguado-Aranda, 2021 (MNCN) • 1♂ – Móstoles {Madrid}, J. Dusmet {leg} / 16-5-30 // MNCN_Ent 301600 (MNCN) • 1♂ – Madrid, 18-8-01 {1901}, G. Schramm {leg} // MNCN_Ent 142907 // SYRPHIDAE, Eristalinae, *Eumerus barbarus* (Coquebert, 1804), Det.: P. Aguado-Aranda, 2021 (MNCN) • 1♂ – Montarco, G. Mercet {leg} // MNCN_Ent 142910 // SYRPHIDAE, Eristalinae, *Eumerus barbarus* (Coquebert, 1804), Det.: P. Aguado-Aranda, 2021 (MNCN) • 1♂ – Madrid, Mercet {leg} // MNCN_Ent 142909 // SYRPHIDAE, Eristalinae, *Eumerus barbarus* (Coquebert, 1804), Det.: P. Aguado-Aranda, 2021 (MNCN) • 2♀♀ – Rivas, Dusmet {leg} // MNCN_Ent 301598; 301601 (MNCN) • 1♀ – El Pardo {Madrid}, Arias Encobet {leg} / 22-VI-1906 // MNCN_Ent 142908 (MNCN) • 1♂ – Sierra de Guadarrama, Dusmet {leg} / ~~6-8-12~~ 23-6-32 // MNCN_Ent 301597 (MNCN) • 1♂ – Madrid, Dusmet {leg} / 6-VI-946 // MNCN_Ent 301604 (MNCN) • 1♂ – Villaviciosa, Dusmet {leg} / 23-5-12 {1912} // MNCN_Ent 142911 // SYRPHIDAE, Eristalinae, *Eumerus barbarus* (Coquebert, 1804), Det.: P. Aguado-Aranda, 2021 (MNCN) • 1♀ – Alcalá, Dusmet {leg} / 4-5-33 // MNCN_Ent 301595 (MNCN) • <u>TUNISIEN</u> • 1♂ (Neotype) – N-Tunesien 27.6.1994, Ghar el Melh Strand, 35 Km SO Bizerte, Leg.: Hauser & Tu-Gha // *Eumerus barbarus* (Coquebert, 1804) ♂, det.: M. Hauser 1996 // Neotype ♂, *Eumerus barbarus* Coquebert, 1804, design J. van Steenis, 2016 {red label} // Zool. Mus. Berlin (ZMHU) {published in van Steenis et al. [5]} • 1♂ – same data as for preceding // *Eumerus barbarus* (Coquebert, 1804) ♂, det.: M. Hauser 1996 // DNA CEUA_S328 (CSCA) {published in van Steenis et al. [5]} • 1♀ – same data as for preceding // *Eumerus barbarus* (Coquebert, 1804) ♀, det.: M. Hauser 1996 // DNA CEUA_S329 (CSCA) {published in van Steenis et al. [5]} • 1♂ – Tunesien, Tabarka, Khathairia, 15.5.1993, leg.: M. Hauser // DNA CEUA_S331 (CSCA) {published in van Steenis et al. [5]} • 1♂ – TUNISIA, S M'Saken, 5km N Sidi Bou Goubrine, 21.V.1999, ~100m asl, 35º36.29´N 10º36.04´E, leg. O. & M. Niehuis // *Eumerus barbarus* (Coquebert, 1804) ♂, det.: M. Hauser 2000 (CSCA) {published in van Steenis et al. [5]} • 1♀ – same data as for preceding // *Eumerus barbarus* (Coquebert, 1804) ♀, det.: M. Hauser 2000 (CSCA) {published in van Steenis et al. [5]} • 1♀ – W-Tunesien, 5 km n El Kef, Tal m. Eryngium, leg. Schmid-Egger, 22.6.1994, Tu-kef // *Eumerus barbarus* ♀ {hand written}, det. Doczkal 1995 (CSCA) {published in van Steenis et al. [5]} • 1♂ – Biserta (Hafenstadt), Tunisia, 13.05.1959, leg.: H. Roer // ZFMK DIP 00017489 // DNA CEUA_S230 (ZFMK).

Eumerus gibbosus **van Steenis, Hauser & van Zuijen, 2017**

New. <u>SPAIN</u> • 1♂ – ESPAÑA, Valencia, Sª Mariola, Bocairent, Font del Mas dels Arbres, campo *Thapsia villosa*, 03-VI-2020, Leg.: Z. Nedeljković // FAUNA IBERICA ERISTALINAE // DNA CEUA_S44 // CEUA00107909 (CEUA-CIBIO) • 1♀ – ESPAÑA, Alicante, Alcoleja, Sierra Aitana, Puerto de Tudons, campo de *Thapsia villosa*, 29-V-2020, Leg.: A. Ricarte // FAUNA IBERICA ERISTALINAE // DNA CEUA_S48 // CEUA00107917 (CEUA-CIBIO) • 1♀ – same locality as for preceding, 06-VI-2020, Leg.: Z. Nedeljković // FAUNA IBERICA ERISTALINAE // DNA CEUA_S49 // CEUA00107915 (CEUA-CIBIO) • 1♀ – same locality and date as for preceding, Leg.: A. Ricarte // FAUNA IBERICA ERISTALINAE // DNA CEUA_S153 // CEUA00107916 (CEUA-CIBIO) • 1♂ – ESPAÑA, Almería, Fondón, Bajo Viaducto Río Andarax, 36º59´13´´N 2º50´39´´W, 4-X-2021, Leg.: I. Ballester-Torres // DNA CEUA_S152 // CEUA00110952 (CEUA-CIBIO).

Eumerus schmideggeri **van Steenis, Hauser & van Zuijen, 2017**

New. <u>TUNISIA</u> • 1♂ – TUN, 8km S Tamerza, 34º20´N 07º56´E, 23.3.2001, leg. Schmid-Egger // ZFMK DIP 00017482 // DNA CEUA_S229 (CSCA).

Revised. <u>TUNISIA</u> • 1♂ (Paratype) – Tunesien, 21.6.2194, Gafsa/Oasengarten, leg. Hauser & Tu-Gaf // coll. Hauser // Paratype ♂, *Eumerus schmideggeri* van Steenis, Hauser & van Zuijen, 2017 {red label} (CSCA) {published in van Steenis et al. [5]}.

***Eumerus sulcitibus* Rondani, 1868**

New. SPAIN • 3♂♂ – ESPAÑA, Valencia, Sª Mariola, Bocairent, Font del Mas dels Arbres, campo *Thapsia villosa*, 12-VI-2021, Leg.: A. Ricarte // CEUA00110930-31, 00110935 (CEUA-CIBIO) • 1♂ – same locality and date as for preceding, Leg.: Z. Nedeljković // CEUA00110936 (CEUA-CIBIO) • 1♂ – ESPAÑA, Alicante, la Vall d'Ebo, 38º48´16.47´´N 0º11´42.97´´O, 546m, campo *Thapsia villosa*, 20-V-2021, Leg.: A. Ricarte // DNA CEUA_S51 // CEUA00107913 (CEUA-CIBIO) • 1♂ – ESPAÑA, Alicante, la Vall de Laguar, Venta del Collao, 38º46´10.91´´N 0º9´4.21´´O, 762m, 26-V-2021, Leg.: A. Ricarte // CEUA00110933 (CEUA-CIBIO) • 1♀ – same data as for preceding // DNA CEUA_S151 // CEUA00110939 (CEUA-CIBIO) • 3♂♂ – ESPAÑA, Madrid, Cercedilla, borde Ctra. M-622, 40º44´10´´N 4º2´15.7´´O, 1200m, 27-VII-2021, Leg.: A. Ricarte // En flores de *Magydaris panacifolia* // DNA CEUA_S154 // CEUA00110937; 00110938; 00110940 (CEUA-CIBIO) • 1♂ – ESP, León, Posada de Valdeón, cerca hotel Cumbres, 43º9´3´´N 4º55´9´´O, 923m, 23-VIII-2021, Leg.: A. Ricarte & Z. Nedeljković // CEUA00110934 (CEUA-CIBIO) • 1♂ – ESPAÑA, León, Crémenes, 28-VIII-2021, 997 m, en *Daucus* sp., Leg.: P. Aguado-Aranda // DNA CEUA_S94 // CEUA00110932 (CEUA-CIBIO) • 3♂♂ – Pelabravo (SA), 15-VII-78, Leg. Mª.A. Marcos // SYRPHIDAE, *Eumerus sulcitibius* Rondani, Det.: Mª.A. Marcos-García / CEUA00017902-04 (CEUA-CIBIO).

Revised. SPAIN • 2♂♂ – Lebeña, SANTANDER, 15-VII-1987, Mª.A. Marcos-García {leg} // SYRPHIDAE, *Eumerus sulcitibius* Rondani, Det.: Mª.A. Marcos-García // CEUA00017906-07 (CEUA-CIBIO) {published in Marcos-García [28]} • 1♀ – same data as for preceding // SYRPHIDAE, *Eumerus sulcitibius* Rondani, Det.: Mª.A. Marcos-García // CEUA00017908 (CEUA-CIBIO) {published in Marcos-García [28]} • 1♂ – Escurial de la Sierra (SA), 17-VI-80, Leg.: Mª.A. Marcos // SYRPHIDAE, *Eumerus sulcitibius* Rondani, Det.: Mª.A. Marcos-García // CEUA00017901 (CEUA-CIBIO) {published in Marcos-García [27]} • 1♂ – El Cabaco (SA), 29-VI-80, Leg.: Mª.A. Marcos // SYRPHIDAE, *Eumerus sulcitibius* Rondani, Det.: Mª.A. Marcos-García // CEUA00017899 (CEUA-CIBIO) {published in Marcos-García [27]} • 1♂ – Montemayor del Río (SA), 29-VI-80, Leg.: Mª.A. Marcos // SYRPHIDAE, *Eumerus sulcitibius* Rondani, Det.: Mª.A. Marcos-García // CEUA00017900 (CEUA-CIBIO) {published in Marcos-García [27]} • 1♀ – Valle de Casares, LEON, 4-VI-87, Mª.A. Marcos-García {leg} // SYRPHIDAE, *Eumerus sulcitibius* Rondani, Det.: Mª.A. Marcos-García // CEUA00017909 (CEUA-CIBIO) {published in Marcos-García [28]} • 1♂ – Font Retura, 900 m, Agres (ALICANTE), 14-30.VIII.01 {Malaise trap}, Leg.: Pérez Bañón, Marcos-García y Rojo / 4913 // CEUA00113513 (CEUA-CIBIO) {published in Mengual [29]} • 1♂ – same locality and leg as for preceding, 30.VIII-2.IX.01 {Malaise trap} / 4917 // CEUA00113514 (CEUA-CIBIO) {published in Mengual [29]} • 1♀ – El Ventorrillo, Madrid, ESPAÑA, 1480 m, Tr. Malaise, 1-6/VI-1990, Nieves & Rey leg. // SYRPHIDAE, *Eumerus sulcitibius* ♀, Det.: Antonio Ricarte // CEUA00107926 (CEUA-CIBIO) {published in Lorenzo et al. [26]} • 1♂ – Cuenca, J.M. Dusmet {leg} // MNCN_Ent 301586 (MNCN) • 1♂ – Sena de Luna (León), Platos, 3-IX-2010, F. Fresno leg. // MNCN_Ent Nº Cat. 57046 (MNCN).
